# Genetic and Environmental Controls on Nitrous Oxide Accumulation in Lakes

**DOI:** 10.1371/journal.pone.0121201

**Published:** 2015-03-10

**Authors:** Jatta Saarenheimo, Antti J. Rissanen, Lauri Arvola, Hannu Nykänen, Moritz F. Lehmann, Marja Tiirola

**Affiliations:** 1 Department of Biological and Environmental Science, University of Jyväskylä, 40014, Jyväskylä, Finland; 2 Lammi Biological Station, University of Helsinki, 16900, Lammi, Finland; 3 Department for Environmental Science, University of Basel, CH-4058, Basel, Switzerland; CAS, CHINA

## Abstract

We studied potential links between environmental factors, nitrous oxide (N_2_O) accumulation, and genetic indicators of nitrite and N_2_O reducing bacteria in 12 boreal lakes. Denitrifying bacteria were investigated by quantifying genes encoding nitrite and N_2_O reductases (*nirS*/*nirK* and *nosZ*, respectively, including the two phylogenetically distinct clades *nosZ*_I_ and *nosZ*_II_) in lake sediments. Summertime N_2_O accumulation and hypolimnetic nitrate concentrations were positively correlated both at the inter-lake scale and within a depth transect of an individual lake (Lake Vanajavesi). The variability in the individual *nirS*, *nirK*, *nosZ*_I_, and *nosZ*_II_ gene abundances was high (up to tenfold) among the lakes, which allowed us to study the expected links between the ecosystem’s *nir*-vs-*nos* gene inventories and N_2_O accumulation. Inter-lake variation in N_2_O accumulation was indeed connected to the relative abundance of nitrite versus N_2_O reductase genes, i.e. the (*nirS*+*nirK*)/*nosZ*_I_ gene ratio. In addition, the ratios of (*nirS*+*nirK*)/*nosZ*_I_ at the inter-lake scale and (*nirS*+*nirK*)/*nosZ*_I+II_ within Lake Vanajavesi correlated positively with nitrate availability. The results suggest that ambient nitrate concentration can be an important modulator of the N_2_O accumulation in lake ecosystems, either directly by increasing the overall rate of denitrification or indirectly by controlling the balance of nitrite versus N_2_O reductase carrying organisms.

## Introduction

Nitrous oxide (N_2_O) is an important greenhouse gas and the single most important ozone destroying chemical [1]. N_2_O in the biosphere is produced as an intermediate molecule in denitrification or nitrifier-denitrification, or as a by-product during nitrification or dissimilatory nitrate reduction to ammonium (DNRA) [2, 3]. The denitrification pathway includes four enzymatically catalyzed reductive steps: nitrate reduction (*nar*), nitrite reduction (*nir*), nitric oxide reduction (*nor*), and nitrous oxide reduction (*nos*) [4]. Reduction of nitrite, where the first gaseous form of fixed nitrogen (N) (i.e. NO) is produced, is catalyzed by two analogous genes: *nirK* and *nirS* genes encoding a copper nitrite reductase and a cytochrome cd1-nitrite reductase, respectively [4]. These two genes prevail in different organisms and their differential distributions in nature seem to be modulated by the redoxconditions, with *nirS* being preferentially expressed under low dissolved oxygen conditions [5, 6]. Recent studies have also revealed that *nos*Z genes encoding N_2_O reductase actually belong to two phylogenetically distinct clades [7, 8], here referred to as *nos*Z_I_ and *nos*Z_II_, which need to be analyzed by separate PCR primer sets. As with *nir* genes, the relative importance of *nos* genes seems to systematically differ between habitats and with environmental conditions [8], yet the exact controls that modulate their relative abundance in nature are uncertain. Some denitrifiers are lacking the *nos*Z gene completely and perform the truncated denitrification pathway, where N_2_O is produced as an end-product [9]. In fact, genome sequencing showed that one third of the cultivated denitrifying bacteria lack the *nos*Z gene [10].

Since denitrifier community structure is likely to have an effect on net N_2_O production and emission [11, 12], denitrifier communities have been studied through the analysis of sequence variation and/or the abundance of *nirS*, *nirK*, and *nosZ* genes in many ecosystems [13, 14, 15, 16, 17]. High availability of nitrate and nitrite has been shown to be conducive to N_2_O accumulation [18, 19], fostering the increase the N_2_O/(N_2_O+N_2_) ratio in the gaseous denitrification products [20, 21]. Such correlations may simply indicate nitrate-induced enhancement of denitrification rates (and thus N2O accumulation), but they may also be the result of microbial community adaptation. Philippot et al. [22], for example, demonstrated that the relative abundance of the *nosZ* gene was a strong predictor of the N_2_O/(N_2_O+N_2_) production ratio.

In soils, microbially produced N_2_O is likely lost to the atmosphere by turbulent diffusive escape. In contrast, in aquatic environments, the diffusivity of gases is much slower (K_z_ values on the order of 10^−5^ to 10^−6^ cm^2^ s^−1^, [23]), reducing diffusive loss rates and improving the N_2_O availability for *nos*Z carrying bacteria. More complete denitrification and lower N_2_O/N_2_ gas emission ratios should, therefore, be expected for the aquatic versus soil environments. Still, lake ecosystems have shown to be important sites of N_2_O emissions [19, 24], and, as in soils, N_2_O production and accumulation in lakes appears to be dependent on the ambient nitrate and oxygen concentrations [25, 26, 24, 27]. Although the importance of lacustrine N_2_O production is well recognized [19, 26], and albeit the fact that benthic denitrifier community structure has been studied in some lakes [28, 29], it is not known whether variations in the accumulation of N_2_O are mostly directly dependent on the environmental conditions, or whether they rather are indirectly constrained by the denitrifying community structure. With some recent exceptions [7, 8] the role of the *nos*Z_II_ clade remained mostly unconsidered in this context.

Here, we evaluated genetic and environmental factors that likely modulate N_2_O production and accumulation in lake ecosystems, especially focusing on the benthic abundance of *nirS*, *nirK*, *nosZ*_I_, and *nosZ*_II_ genes during the summertime N_2_O accumulation period. Anticipating close links between nitrate concentrations and the N_2_O accumulation, we hypothesized 1) that high hypolimnetic nitrate concentrations would decrease the relative abundance of the *nosZ* genes (i.e., increase the *nir*/*nos* ratio) within lacustrine sediments, and 2) that higher *nir*/*nos* ratios would lead to enhanced N_2_O accumulation. The linkage between benthic denitrification gene frequency and N_2_O accumulation was assessed in an inter-lake study of 12 boreal lakes in southern Finland, pooling the lakes into two groups based on their hypolimnetic nitrate concentrations (high-NO_3_^−^-lakes and low-NO_3_^−^-lakes). In addition, denitrification gene abundance and N_2_O accumulation was investigated along a littoral-to-pelagic transect in a large stratified lake (Vanajavesi) with relatively high hypolimnetic nitrate levels (24.0−44.9 μmol l^−1^).

## Results

### Comparison of denitrification genes in high- versus low-nitrate lakes

Considerable inter-lake variation was observed with regards to the nitrate (0.4−79.1 μmol l^−1^), ammonium (0.6−61.4 μmol l^−1^), and oxygen (1.9−333.4 μmol l^−1^) concentrations (S1 Table). The lakes were classified into two groups based on their nitrate concentration, which generally reflected land use in the catchment area: high-NO_3_^−^-lakes comprised lakes mostly with extensive agricultural activity in their catchment area and one urban lake (Jyväsjärvi), while low-NO_3_^−^-lakes included lakes mostly with little agricultural land in their catchment area. Other environmental parameters did not differ significantly between the two groups (Table 1).

**Table 1:** Environmental parameters (mean and SE) for high-NO_3_^−^-lakes (n = 6) and low-NO_3_^−^-lakes (n = 6), and results of a t-test or Mann-Whitney U-test* comparing the oxygen, nitrate, ammonium and phosphate concentrations, temperature, catchment field area (ha), averaged N_2_O_excess_ concentrations, and maximum observed N_2excess_ concentrations between the two lake groups.

	O_2_ (μmol l^−1^)	NO_3_ (μmol l^−1^)	NH_4_^+^ (μmol l^−1^)	PO_4_^−^ (μmol l^−1^)	T (C°)	Field area (ha)	N_2_O_excess_ (μmol m^−3^)	N_2excess_ (μmol l^−1^)
**High-nitrate lakes**								
Mean	101.15	39.30	15.48	0.15	11.41	37990	18.14	5.55
(±SE)	(±51.30)	(±8.76)	(±8.55)	(±0.02)	(±1.49)	(±35870)	(±4.97)	(±0.79)
**Low-nitrate lakes**								
Mean	77.72	0.64	34.67	0.08	15.78	500	1.36	1.12
(±SE)	(±35.77)	(±0.11)	(±17.68)	(±0.03)	(±2.42)	(±245)	(±1.50)	(±0.47)
**Pairwise test results**								
High vs. low nitrate	high = low	high > low	high = low	high = low	high = low	high > low	high > low	high > low
*P*	ns	0.007	Ns	ns	ns	0.012*	0.001	0.001

**Fig 1:**
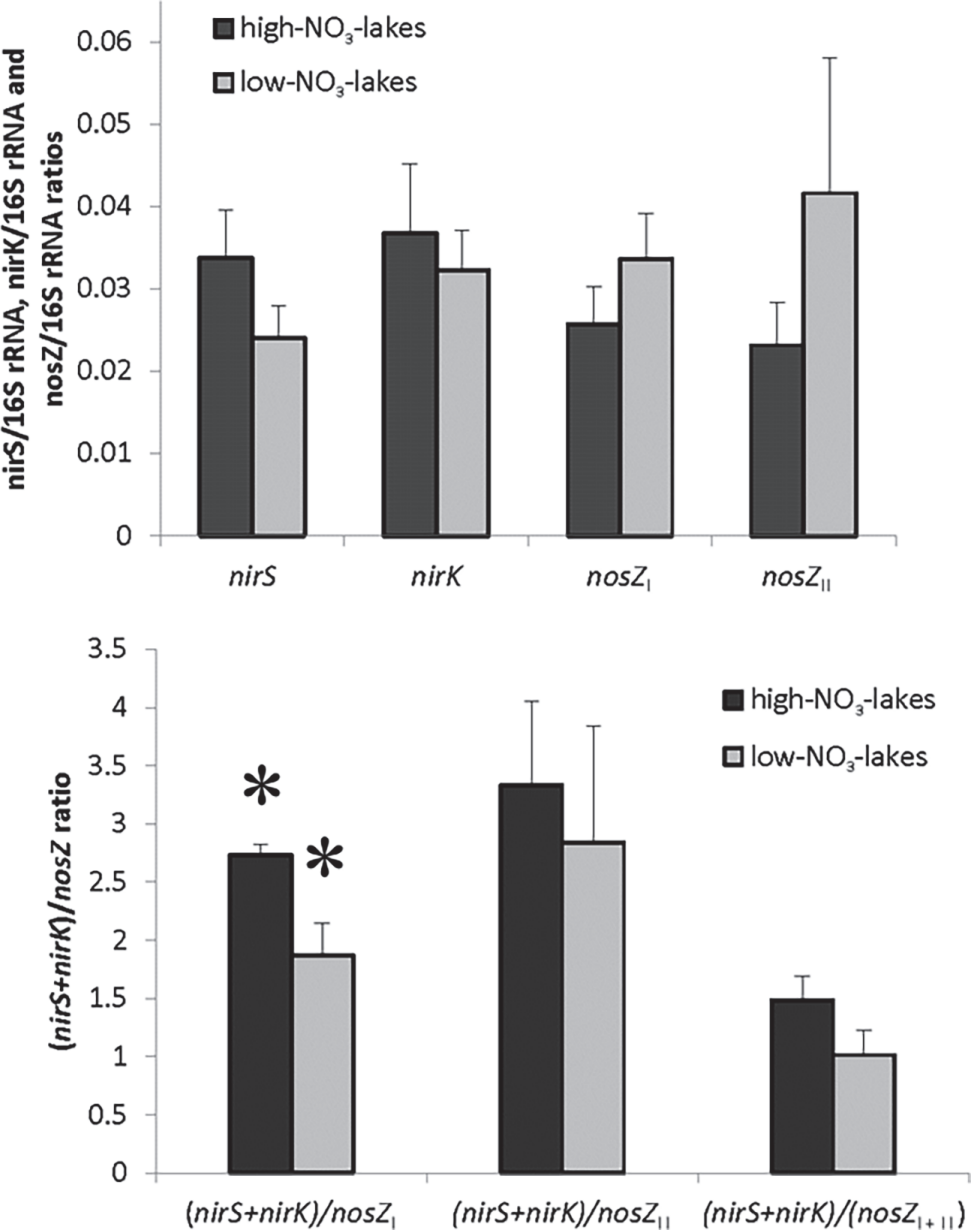
Abundance of *nir*S, *nir*K, *nos*Z_I_, and *nos*Z_II_ genes relative to the amount of 16S rRNA genes (A), and ratios of *nir* and *nos* genes in sediments of lakes with high and low nitrate concentrations (high-NO3^−^-lakes and low-NO3^−^-lakes) (B). _*_ = significantly different between the two lake groups (Mann-Whitney U-test, p = 0.006).

Throughout the studied lakes, the abundances of *nirS*, *nirK*, *nosZ*_I_, and *nosZ*_II_ relative to 16S rRNA genes varied between 0.6−12.9% (Table 2), and the gene copy numbers ranged between 4.8 and 580 per ng of DNA (S2 Table). The ratio of *nir*S/*nir*K ranged between 0.5−2.0 (average 1.0), and the ratio of *nos*Z_I_/*nos*Z_II_ varied between 0.5−5.7 (average 1.9). Neither environmental factors (oxygen, temperature, nitrate concentration) nor N_2_O accumulation showed any significant correlation with the gene abundance, gene copy numbers, or with *nir*S/*nir*K or *nos*Z_I_/*nos*Z_II_ gene ratios (Pearson correlations, p values >0.05). The relative proportion of the previously unaccounted *nosZ*_II_ gene was of a similar magnitude as that of *nosZ*_I_, but showed a markedly higher inter-lake variability (Table 2). Although not statistically significant, *nosZ*_I_ and *nosZ*_II_ seemed slightly more abundant in the low-NO_3_^−^ group of lakes, while *nirS* and *nirK* seemed less abundant, (Fig. 1A). The (*nirS*+*nirK*)/*nosZ*_I_ ratio was higher in high-NO_3_^−^-lakes than in low-NO_3_^−^-lakes (Fig. 1B). In addition, the (*nirS*+*nirK*)/*nosZ*_I_ gene ratio correlated positively with the estimated net N_2_O production, as well as with nitrate and phosphate concentrations (Table 3.). As for (*nirS*+*nirK*)/*nosZ*_II_ and (*nirS*+*nirK*)/(*nosZ*_I_+*nosZ*_II_), we also observed a tendency for higher ratios in the high-NO_3_^−^-lakes compared to low-NO_3_^−^ lakes (Fig. 1B). However, correlation between nitrate and (*nirS*+*nirK*)/(*nosZ*_I_+*nosZ*_II_) was only weakly significant (p = 0.06) (Table 3).

**Table 2:** Copy numbers (mean ±SE) of *nirS*, *nirK*, *nosZ*_I_, and *nosZ*_II_ gene amplicons as percentages of 16S rRNA gene copy numbers (nd, no data).

Inter-lake comparison	Denitrification gene (% of 16S rRNA gene)				High-nitrate/
					Low-nitrate
	*nirS*	*nirK*	*nosZ*_I_	*nosZ*_II_	
Pääjärvi	5.5	7.9	4.7	2.1	High
(±SE)	(±0.25)	(±0.26)	(±0.34)	(±0.07)	
Mommilanjärvi	4.5	2.3	2.5	2.4	High
(±SE)	(±0.28)	(±0.26)	(±0.24)	(±0.18)	
Ormajärvi	3.6	3.9	2.4	2.1	High
(±SE)	(±0.09)	(±0.34)	(±0.08)	(±0.02)	
Vanajavesi	2.9	3.2	2.4	1.3	High
(±SE)	(±0.31)	(±0.28)	(±0.32)	(±0.14)	
Jyväsjärvi	3.0	3.4	2.5	5.2	High
(±SE)	(±0.08)	(±0.24)	(±0.15)	(±0.22)	
Suolijärvi	0.9	1.3	0.9	1.5	High
(±SE)	(±0.38)	(±0.21)	(±0.19)	(±0.10)	
Ekojärvi	1.2	2.5	2.1	2.9	Low
(±SE)	(±0.07)	(±0.31)	(±0.23)	(±0.19)	
Kataloistenjärvi	4.4	5.2	3.9	1.2	Low
(±SE)	(±0.20)	(±0.4)	(±0.32)	(±0.24)	
Teuronjärvi	2.5	3.0	2.8	1.3	Low
(±SE)	(±0.28)	(±0.28)	(±0.29)	(±0.04)	
Kyynäröjärvi	2.5	3.5	2.9	2.5	Low
(±SE)	(±0.25)	(±0.26)	(±0.09)	(±0.07)	
Kastanajärvi	1.9	1.4	6.1	12.9	Low
(±SE)	(±0.04)	(±0.05)	(±0.25)	(±0.25)	
Lehee	2.0	3.8	2.3	4.1	Low
(±SE)	(±0.21)	(±0.22)	(±0.12)	(±0.26)	
Intra-lake depth transect					
Vanajavesi2	4.9	2.4	2.1	1.8	
(±SE)	(±0.28)	(±0.20)	(±0.28)	(±0.08)	
Vanajavesi3	6.2	3.5	3.4	2.4	
(±SE)	(±0.33)	(±0.32)	(±0.43)	(±0.09)	
Vanajavesi4	4.8	3.6	3.4	1.7	
(±SE)	(±0.26)	(±0.32)	(±0.36)	(±0.05)	
Vanajavesi5	3.3	4.2	2.9	1.4	
(±SE)	(±0.29)	(±0.30)	(±0.22)	(±0.10)	
Vanajavesi6	2.4	4.2	2.4	0.6	
(±SE)	(±0.23)	(±0.27)	(±0.26)	(±0.13)	
Vanajavesi7	2.9	3.2	2.4	1.3	
(±SE)	(±0.31)	(±0.28)	(±0.32)	(±0.14)	
Vanajavesi8	3.1	2.2	1.1	nd	
(±SE)	(±0.20)	(±0.19)	(±0.14)		

**Table 3:** Correlations of functional gene ratios and accumulated N_2_O and N_2_ gas concentrations with environmental parameters in the inter-lake dataset. Correlation coefficients with 0.01 < p < 0.05 and p < 0.01 are written in normal text and **bold**, respectively.

	**Gene ratios**			**Gas accumulation measurements**		
	(*nirS+nirK*)/*nosZ*_*I*_	(*nirS+nirK*)/*nosZ*_*II*_	(*nirS+nirK*)/(*nosZ*_*I*_ *+*_*II*_)	N_2_O_excess_ (μmol m^−3^)	N_2_O production (μmol N m^−2^ d^−1^)	N_2excess_ (μmol l^−1^)
O_2_ (μmol l^−1^)	-	-	-	-	-	-
NO_3_ (μmol l^−1^)	**0.78**	-	(0.55)*	0.66	**0.74**	0.58
NH_4_^+^ (μmol l^−1)^	-	-	-	-	-	-
PO_4_^−^ (μmol l^−1^)	0.67	-	-	-	-	-
T (C°)	-	-	-	-	-	-
N_2_O_excess_ (μmol m^−3^)	0.61	-	-	**1**	**0.96**	**0.80**

* Marginally significant (p = 0.06)

### N_2_O and N_2_ accumulation in high- versus low-nitrate lakes

During the summer sampling (late July), most of the study lakes were oversaturated with respect to N_2_O (i.e. the depth-integrated mean N_2_O_excess_ was >0). N_2_O_excess_ in the water column varied between 0.9−37.1 nmol l^−1^ (11−337% oversaturation). The highest N_2_O_excess_ concentrations were observed either in near-bottom waters of the lakes or, in the case of stratified lakes (five lakes were stratified with regards to oxygen and displayed an anoxic hypolimnion), at the oxic-anoxic interface within the water column (S1 Fig.). Maximum N_2excess_ concentrations measured using membrane inlet mass spectrometry (MIMS) were generally slightly higher than the equilibrium concentration at given temperatures (<2% oversaturation). N_2excess_ was significantly higher in high-NO_3_^−^ lakes than in low-NO_3_^−^ lakes (Table 1) and correlated with nitrate concentrations (Table 3). Moreover, the depth-integrated N_2_O_excess_ concentrations (0−20.3 μmol m^−3^) and net N_2_O production rates (0−11.2 μmol N m^−2^ d^−1^) estimated from the N_2_O concentration profiles were significantly higher in high-NO_3_^−^-lakes than in low-NO_3_^−^-lakes (Table 1), and both correlated with NO_3_^−^ concentration (Table 3). Maximum N_2excess_ concentrations were found to correlate with the depth-integrated N_2_O_excess_ concentration (Table 3).

### Denitrification genes and N_2_O accumulation in Lake Vanajavesi

In Lake Vanajavesi, hypolimnetic temperature and oxygen concentrations were tightly correlated, indicating the effect of thermal water column stratification on the vertical distribution of dissolved oxygen (correlation r = -0.98 and p = 0.000). Sampling sites 1−3 (water depths 2−6 m) were fully aerated, sites 4−6 (water depths 8−12 m) displayed lower oxygen concentrations, and the two deepest sampling sites (water depths 14 and 16 m) were anoxic at the bottom of the hypolimnion (S3 Table). Nitrate concentrations (24.0−44.9 μmol l^−1^) were consistently high at all sampling sites, whereas ammonium (1.1−57.4 μmol l^−1^) and phosphate (0.03−0.7 μmol l^−1^) concentrations displayed strong variability between strongly oxygen-depleted and oxygen-replete conditions (S3 Table).

**Fig 2:**
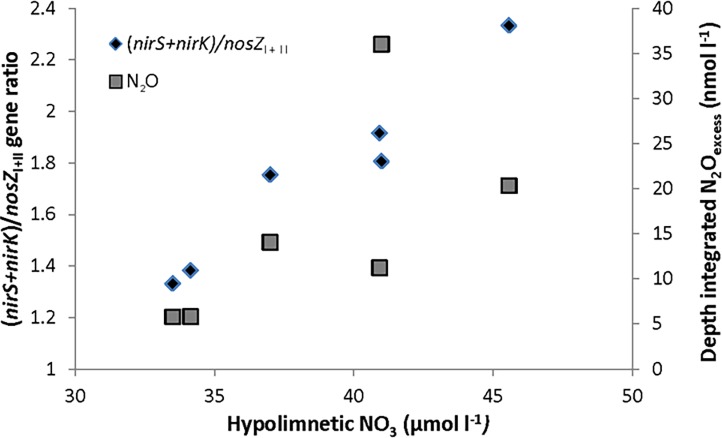
Relationship between hypolimnetic nitrate concentration and the sedimentary (0–2cm) (*nirS+nirK*)/*nosZ*_I+II_ gene ratio (r = 0.98 and p = 0.001), and depth-integrated N_2_O_excess_ (r = 0.89 and p = 0.02) in Lake Vanajavesi.

The relative abundances of *nir*S, *nir*K, *nos*Z_I_, and *nos*Z_II_ genes in Lake Vanajavesi varied between 0.6 and 6.2% of the total 16S rRNA genes (Table 2), with *nos*Z_I_ or *nos*Z_II_ being the least abundant of the denitrifying genes at all sites. In contrast to observation at the inter-lake scale (where nitrate concentrations were generally lower and more variable), we observed a strong positive correlation between nitrate concentrations and the (*nirS*+*nirK*)/*nosZ*_I+II_ ratio (r = 0.98 and p = 0.001) (Fig. 2). The correlation with either *nos*Z_I_ or *nos*Z_II_ only was not significant (p > 0.05).

At all sampling sites, essentially the entire water column was oversaturated with respect to equilibrium N_2_O concentrations (S2 Fig.). The N_2_O profiles of Sites 1, 2, and 3 indicated a homogenized water column, with an equal degree of oversaturation throughout. At the deeper Sites 4, 5, and 6, a markedly higher N_2_O oversaturation was observed at the bottom of the lake. The degree of N_2_O oversaturation was even higher at the oxic-anoxic interface in the water column of Sites 7 and 8 (S2 Fig.). Depth-integrated N_2_O_excess_ varied between 5.7−36.0 nmol l^−1^ (62−337% oversaturation) and correlated positively with the nitrate concentration in Lake Vanajavesi (r = 0.89 and p = 0.02) (Fig. 2). A negative correlation was observed with respect to the oxygen concentration (r = -0.90 and p = 0.002) and temperature (r = -0.95 and p < 0.001).

## Discussion

To our knowledge, this is the first study combining N_2_O measurements and molecular analyses of denitrification genes in lake ecosystems. This is also the first time that the abundance of *nirS* and *nirK* genes together with both clades of *nosZ* genes were investigated in freshwater sediments. The total *nir*/*nos* ratio was above 1:1 in nearly all study lakes, indicating that the microbial community had a higher potential to produce N_2_O than to reduce it. This implies that the accumulation of N_2_O is linked to genetic factors.

All the studied denitrification genes (*nir* and *nos* variants) were present in the lake sediments, although their abundance largely varied among the lakes and along the Vanajavesi transect. The qPCR results also revealed that *nosZ*_II_ genes are as frequent as the canonical *nosZ*_I_ genes in the freshwater sediments, which emphasizes the need to further study the ecology of *nosZ*_II_ encoding organisms in future studies. The relatively high abundance of individual *nirS*, *nirK*, *nosZ*_I_, and *nosZ*_II_ genes highlights the important biogeochemical role of denitrification in boreal lake sediments. For comparison, the abundance of individual denitrification genes *nirS*, *nirK*, and *nosZ* have previously been found to range between 0.5 and 6.8% of the 16S rRNA gene abundance in various soil and sediment samples [14, 21, 30, 31]. Bioavailability of copper (Cu) and iron (Fe) can control the expression and activity of nitrite and nitrous oxide reductases. While *nirK* and *nosZ* are copper-containing reductases, *nirS* is an iron containing cd1-type reductase. Possible Cu limitation may lead to *nirS* dominance and, thus, to increased N_2_O accumulation. Unfortunately, data on Fe and Cu concentrations were not available, and we cannot fully exclude Cu versus Fe limitation as a controlling factor in N_2_O accumulation in the study lakes. Yet, the equal abundance of *nirS* and *nirK* genes does not suggest any adaptation of the microbial community to Cu limitation.

Data on the *nirS*/*nirK* gene ratios in lakes are rare. The only study we know of in this context is by Martins et al. [31], who reported that *nirS* genes were more abundant than *nirK* genes in sediments of freshwater lakes on the Azores. In contrast, the average *nirS*/*nirK* gene ratio observed in this study was 1:1. Different from the subtropical lakes studied by Martins et al. [31], boreal lakes experience seasonal variations in redox and other physico-chemical conditions, which may increase the diversity of ecological niches and prevent certain microbial ecotypes from dominating an ecosystem. Since the distribution of *nirS* and *nirK* genes is phylogenetically scattered [10], the ratio of these two evolutionarily separate, but functionally equivalent, nitrite reductase gene types does not necessarily reflect the dominance of one taxonomical group over another as a function of environmental conditions. Instead, the relatively strong variability in the *nirS* and *nirK* gene ratio between the existing studies highlights the need to quantify both genes when studying the factors affecting N_2_O accumulation. Although the *nir*/*nos* ratio at the DNA level does not necessarily correspond to the respective ratios at the level of mRNA transcripts or enzyme molecules on short-term time scales, it may indicate longer-term genetic adaptation, which was the focus of this study.

When comparing lakes at different spatial scales and between various geographical regions, denitrification rates have shown a clear positive correlation with nitrate availability [32]. This correlation was further corroborated by the observed co-variation of NO_3_^−^ and N_2excess_ in the lakes studied here. Our study also showed the linkage between NO_3_^−^ concentration and N_2_O accumulation, which is in agreement with previous work in boreal lakes [18]. Based on previously published N_2_ production rates for five of the lakes in this study [29, 32] (unpublished results), the N_2_O production rates reported here correspond to 0.2−1.7% of the total gaseous N production (N_2_O/(N_2_+N_2_O) ratio). These values fall within the range of previously reported estimates (0.1–4.1%) for freshwater systems [33]. Besides total denitrification rates, it is the balance between nitrite reduction and N_2_O reduction which controls the build-up of N_2_O. This balance has been shown to be sensitive to changes in redox conditions [34]; however, the role of longer-term nitrate availability in modulating this balance is uncertain. NO_3_^−^ is generally the preferred electron acceptor for the denitrifying community when compared to N_2_O (except for some *nosZ*_II_ carrying organisms, see the discussion below). Hence, when the competition for nitrate is tighter, reduction of N_2_O becomes a more feasible trait for the heterotrophic micro-organisms [35].

At the inter-lake scale, *nir*/*nosZ*_I_ ratios correlated with the nitrate concentrations and N_2_O_excess_. These correlations suggest that the denitrifying communities were adapted to varying nitrate levels within the lake and that they control the ratio of N_2_O production versus reduction. Moreover, both at the inter-lake scale and within the Lake Vanajavesi transect the combined *nir*/*nos* ratio (i.e. [*nirS*+*nirK*]/*nosZ*_I+II_) correlated with ambient nitrate. In contrast, the *nir*/*nosZ*_I_ ratio did not display any statistically significant correlation with (the less variant) nitrate concentration in Lake Vanajavesi. This apparent difference with regards to the role of *nosZ*_I_ and *nosZ*_II_ may be related to the known genetics of *nosZ*_II_ carrying organisms. The N_2_O reductase *nosZ*_I_ has only been found for *Alpha*-, *Beta*-, and *Gammaproteobacteria* and some archaea, whereas *nosZ*_II_ reductases may be common in a wider range of bacterial and archaeal phyla [7, 8]. While most of the typical *nosZ*_I_-harboring microbes have the complete set of denitrification genes, less than half of the known *nosZ*_II_-carrying microorganisms possess genes of the “upstream” denitrification steps, and *nosZ*_II_-type reductase was thus named as “non-denitrifier nitrous oxide reductase” [7]. As a consequence, many of the *nosZ*_II_-carrying microbes are incapable of using nitrate (or nitrite) as an electron acceptor, and are, therefore, less affected by ambient nitrate availability. The variable prevalence of denitrifying versus non-denitrifying *nosZ*_II_ subsets may explain the above-described differences in the correlation analyses between the inter-lake and intra-lake studies (genetic relationships versus NO_3_^−^ levels).

Although it has been shown that denitrification is the major N_2_O source in lake ecosystems [19, 27], it is likely that nitrifiers (i.e. ammonium oxidation and nitrifier-denitrification) also contribute to N_2_O production in these environments. In the lake transect, where sampling sites where characterized by different hypolimnetic oxygen regimes, N_2_O accumulation patterns were clearly linked to oxygen concentration. Concentration of N_2_O peaked near the oxic-anoxic interface, which was located either in the sediment surface or in the water column. This could be due to O_2_ availability just above the oxic-anoxic interface, which would increase N_2_O production via nitrification [36, 37]. On the other hand, the presence of O_2_ even at low levels likely inhibits N_2_O reduction compared to other reduction steps in denitrification [37]. Therefore, truncated denitrification would also lead to observed accumulation patterns of N_2_O, with concentration maxima in the vicinity of the redox transition zones. The lack of N_2_O accumulation in the anoxic water layers of the lakes further supports the notion that stable anoxic conditions are conducive to full denitrification to N_2_, while microaerophilic conditions would rather support truncated denitrification and/or slowed nitrous oxide reduction. In addition, dissimilatory nitrate reduction to ammonium (DNRA), in which N_2_O can also be formed as a by-product [38], competes with denitrification for nitrate. The most important factor controlling competition between these two processes appears to be the C:N ratio [39, 40], where high ratios favors DNRA over denitrification. In addition, the supply of nitrate relative to nitrite and microbial generation time are identified as key environmental factors in controlling whether nitrate is reduced to nitrogen gas in denitrification, or retained in the ecosystem as ammonium in DNRA [41]. In our study lakes, the C:N ratio of sediment organic material varied between 9 and 27 (on average 17.7), and thus DNRA may have had some role on NO_3_^−^ reduction. However, the actual contribution of N_2_O production by organisms carrying out DNRA in lake ecosystems is currently unresolved.

This study provided putative evidence for the control of both denitrifier gene composition and N_2_O accumulation by nitrate concentration. This suggests that N_2_O emissions from denitrification would be modulated by nitrate-induced changes in the denitrifier communities. In turn, the study indicates that recent increases in the land-based and atmospheric anthropogenic nitrogen loadings from agriculture and energy production may have caused shifts in the lacustrine denitrifier communities as well as stimulated N_2_O emissions from lake ecosystems.

## Experimental Procedures

### Study sites and the sampling procedure

The study lakes are located within the same region in southern Finland (61°01−61°52 N and 25°02−24°09 E), except Lake Jyväsjärvi which is located 150 km north of the other lakes (62°13 N and 25°44 E) (S1 Table). The lakes are located on state land with open access, thus no permits were required for collection of samples. Further, the locations are not protected in any way and the study did not involve endangered or protected species. All the study lakes were sampled in July 2011. The lakes were chosen to cover a wide variety of lake characteristics: size (surface area 25−12000 ha), maximum depth (2−85 m), and nutrient concentrations (S1 Table). All the study lakes are ice-covered from November until the beginning of May. We divided the selected lakes into two groups based on their hypolimnetic nitrate concentrations. High-NO_3_^−^-lakes (n = 6) comprised lakes with NO_3_^−^ concentrations between 15.7−79.4 μmol l^−1^ and low-NO_3_^−^-lakes (n = 6) included lakes with NO_3_^−^ concentrations between 0.6−1.5 μmol l^−1^ (S1 Table).

Depths of the sampling sites were recorded with an echo-sounder (S1 Table) and the water samples were taken with a Limnos tube sampler (height 30 cm, volume 2.1 l). Water samples for gas analyses were collected at ca. 0.5 m, 1 m, 3 m, and 5 m above the lake bottom (if the lake was deep enough) and below/under the surface (0.5 m water depth). Three replicates (30 ml) were taken from each depth for N_2_O concentration measurements in 60 ml polypropylene syringes, which were closed with three-way stopcocks after removing any gas bubbles, and transported to the laboratory on ice. Nitrogen gas (N_2_) samples for membrane inlet mass spectrometry (MIMS) measurements were taken in 12 ml borosilicate glass tubes (six replicates) with screw-capped butyl rubber septa (Labco Ltd.). We allowed water overflow for at least three volumes to avoid atmospheric contamination, and samples with air bubbles were discarded. Microbial processes in borosilicate glass tubes were stopped by adding 100 μl ZnCl through the septum with a needle under water. Water for nutrient analyses were collected in 1-L bottles from the near-bottom waters of the lakes and all samples were transported to the laboratory on ice. Sediment core samples for analyses of the denitrifier communities were collected in all of the lakes using a mini gravity corer with plexiglass tubes (ø = 3.5 cm).

Water column profiles of temperature and oxygen concentrations were measured *in situ* using a portable field meter (YSI model 58, Yellow Springs Instruments). Dissolved inorganic phosphorus [42], nitrate [43], and ammonium [44] were determined with a flow injection analyzer using standard methods (QuikChem 8000) from filtered (0.2 mm filter; Millipore) water samples.

### Quantification of *nirS*, *nirK*, and *nosZ* genes

Sediment samples were collected from the surface layer (0–2 cm) of the sediment cores and freeze-dried for further use (Alpha 1–4 LD plus, Christ). DNA extraction was performed from 0.03 g of dry sediment using the bead-beating and phenol-chloroform extraction protocol of Griffiths et al. [45]. Two extractions were made from each site. The DNA concentrations were measured with a Qubit 2.0 Fluorometer (Invitrogen) and the DNA concentration of each sample was adjusted to yield a concentration of 10 ng μl^−1^.

For qPCR quantification of the *nirK*, *nirS*, *nosZ*_I_, and *nosZ*_II_ genes, partial 16S rRNA was used as a reference gene, and commonly used primers were selected from previous studies (S4 Table). Amplification of qPCR and fluorescent data collection was carried out with a Bio-Rad CFX96 thermal cycler (Bio-Rad Laboratorios) in a reaction mixture of 0.5 μM of each primer for the selected target gene (except for *nos*Z_II_ 1 μM of each primer), 10 μl 2XiQ SYBR Green supermix (BioRad), 1 μl of DNA (10 ng), and PCR-grade water (Fermentas) to yield a total volume of 20 μl. Three replicate qPCR amplifications were performed for each sample.

The PCR procedure for 16S rRNA included an initial denaturation step at 95°C for 15 min and 40 cycles of amplification (95°C for 20 s, 53°C for 35 s and 72°C for 70 s). Finally, an increase of 0.5°C s^−1^ from 65 to 95°C was performed to obtain the melting curve analysis of PCR products. The thermal cycling conditions for other genes were the same as the one just described, except that the annealing temperature was 55°C for *nirS*, 60°C for *nirK* and *nosZ*_I_, and 54°C for *nosZ*_II_. Standard curves were constructed from PCR amplicons extracted from agarose gel with a BioRad Gel Extraction Kit (BioRad). Amplicons were re-amplified and the resulting products were purified with Agencourt AMPure XP (Beckman Coulter). A dilution series of 10^7^–10^2^ gene copies were used as standards in each qPCR run. Gene abundances were calculated as relative abundances from the abundance of the reference gene (16S rRNA). Replicate results were averaged (n = 6) and standard errors were calculated. Inhibition was tested from the dilution series (1, 1^–10^ and 1^–100^) and no inhibition was detected.

### N_2_O gas concentrations

N_2_O samples were analyzed according to Maljanen et al. [46] with a gas chromatograph (Agilent 6890N, Agilent Technologies) equipped with an auto sampler (Gilson) and an electron capture detector (ECD). The N_2_O samples were processed according to Bellido et al. [47], and two replicates from each depth were measured. N_2_O equilibrium concentrations were calculated based on Henry’s law (modified from IPCC Fourth Assessment Report: Climate Change 2007 and [48]). Concentration of N_2_O accumulated due to microbial reactions (N_2_O_excess_) was calculated from the difference between observed N_2_O concentration and the calculated equilibrium concentration. The overall amount of accumulated N_2_O per square meter was estimated from integration of the N_2_O_excess_ concentration profiles, and the depth-integrated N_2_O_excess_ per m^3^ was obtained by division through the water depth at the sampling site. All study lakes undergo complete spring mixing after ice-off (with equilibrium concentrations throughout the water column). Assuming cumulative N_2_O production in the hypolimnion, with low atmospheric exchange after the mixing period, net N_2_O production rates can be estimated according to Mengis et al. [25] (with slight modifications) by dividing the amount of accumulated N_2_O per square meter by the number of days since ice-off (i.e. the onset of water column stratification in early May) to the sampling date (end of July). These estimates need to be considered conservative, as at least some turbulent diffusive loss to the atmosphere is indicated by the partial N_2_O pressure gradient between surface water and the atmosphere (see S1 Fig.).

### Natural N_2_ gas concentrations

N_2_/Ar gas concentration ratios were determined using membrane inlet mass spectrometry (MIMS) as described in Kana et al. [49]. Equilibrium concentrations were calculated according to Weiss [50]. N_2excess_ was then calculated from N_2_/Ar ratio in the sample divided by the N_2_/Ar ratio at equilibrium for a given temperature.

### Statistical analyses

Data analyses were conducted using PASW 18.0 (PASW Statistics 18, Release Version 18.0.0, SPSS 2009). The normality assumption was tested with the Shapiro-Wilks test. In our dataset, the effect of nitrate concentration on process parameters and denitrifier communities was specifically addressed by comparing high-NO_3_^−^-lake and low-NO_3_^−^-lake data either using independent samples t-test (normally distributed variables) or Mann-Whitney U-test (non-normally distributed variables). In addition, correlation analysis (Pearson or Spearmann correlation) was performed to study potential relationships among environmental parameters (NO_3_^−^ concentration, oxygen concentration, ammonium concentration, phosphorus concentration, depth, gene abundances, and N_2_O concentrations.

## Supporting Information

S1 Fig Vertical profiles of measured N_2_O concentrations, calculated N_2_O equilibrium concentrations, oxygen concentrations, and temperatures in different lakes. The grey line indicates the respective oxic-anoxic interface.
(TIF)

S2 Fig Vertical profiles of measured N_2_O concentrations, calculated N_2_O equilibrium concentrations, oxygen concentrations, and temperatures at the different sampling sites along the depth transect of Lake Vanajavesi. The grey line indicates the oxic-anoxic interface.
(TIF)

S1 Table Study site information, hypolimnetic nutrient concentrations, and oxygen status of the study lakes. (DOCX)

S2 Table Gene copy numbers of *nirS*, *nirK*, *nosZ*_I_, and *nosZ*_II_ gene amplicons per ng of DNA (nd, no data). (DOCX)

S3 Table Water temperature and pH, as well as nutrient and oxygen concentrations at various sampling sites in Lake Vanajavesi. (DOCX)

S4 Table Gene-specific primer pairs used in the qPCR assays. (DOCX)
