# Supplementary figures and images for: Genetic and Environmental Controls on Nitrous Oxide Accumulation in Lakes
Source: PLoS One. 2015 Mar 10;10(3):e0121201. doi: 10.1371/journal.pone.0121201 (PMC4355481; doi:10.1371/journal.pone.0121201)

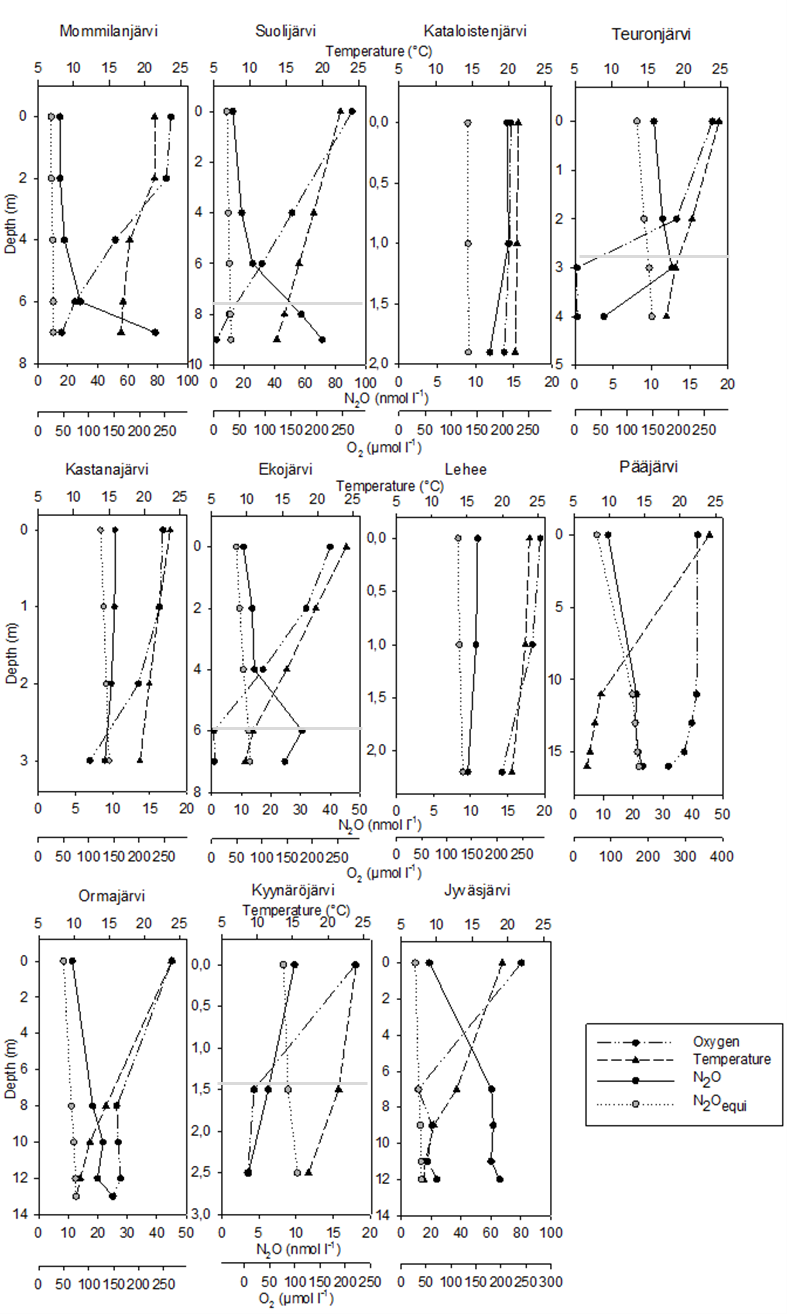

Supplement: S1 Fig — The grey line indicates the respective oxic-anoxic interface. (TIF) [file pone.0121201.s001.tif]

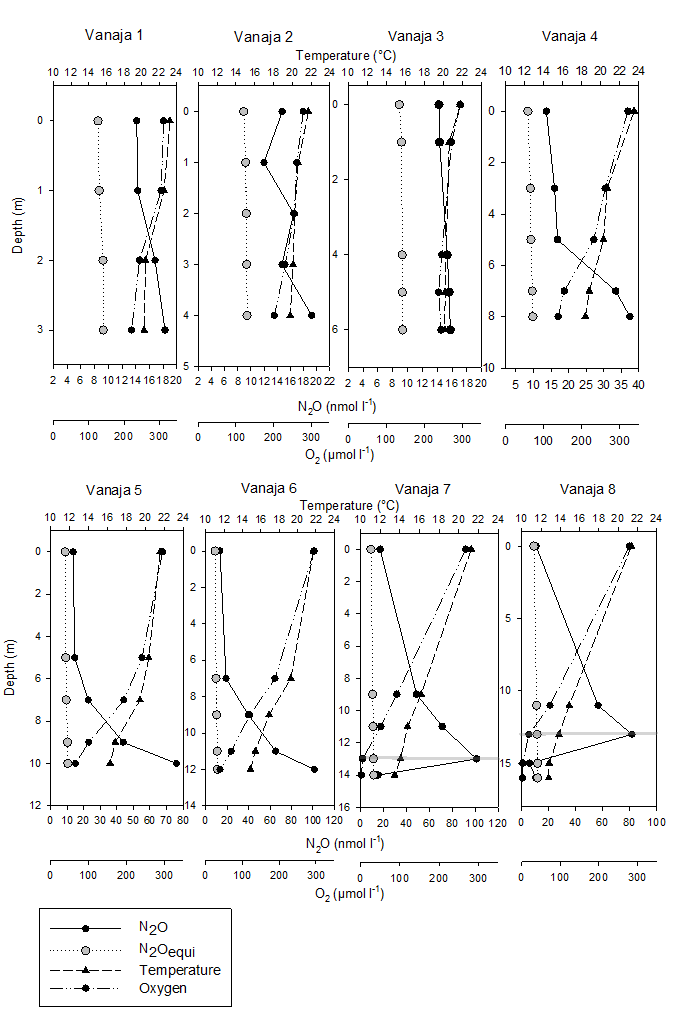

Supplement: S2 Fig — The grey line indicates the oxic-anoxic interface. (TIF) [file pone.0121201.s002.tif]
