# Supplementary material for: Genetic and Environmental Controls on Nitrous Oxide Accumulation in Lakes
Source: PLoS One. 2015 Mar 10;10(3):e0121201. doi: 10.1371/journal.pone.0121201 (PMC4355481; doi:10.1371/journal.pone.0121201)
Supplement: S1 Table — (DOCX) [file pone.0121201.s003.docx]

Table S1. Study site information, hypolimnetic nutrient concentrations, and oxygen status of the studied lakes.

| Lake | Area (ha) | Catchment field area (ha) | Max Depth (m) | Sampling depth (m) | Sampling site | Temperature  °C | NO_3_  (µmol l^-1^) | PO_4_ (µmol l^-1^) | NH_4_ (µmol l^-1^) | High-nitrate / Low-nitrate | O_2_  (µmol l^-1^) |
| --- | --- | --- | --- | --- | --- | --- | --- | --- | --- | --- | --- |
| Pääjärvi | 1344 | 3816 | 85 | 16 | N 61º03´05.8``  E 25º04`43.1`` | 6.8 | 79.1 | 0.2 | 1.7 | High | 333.4 |
| Mommilanjärvi | 342 | 1585 | 8 | 7 | N 61º52`49.6``  E 25º2`30.2`` | 16.6 | 42.6 | 0.2 | 13.0 | High | 46.9 |
| Ormajärvi | 653 | 2067 | 32 | 13 | N 61º05´43.0``  E 24º59`16.5`` | 10.3 | 34.5 | 0.1 | 5.2 | High | 147.5 |
| Vanajavesi | 12 000 | 181461 | 24 | 16 | N 61º09´03.2``  E 24º16`11.1`` | 13.5 | 32.1 | 0.1 | 57.4 | High | 1.9 |
| Jyväsjärvi | 330 | 0* | 25 | 13.5 | N 62º13´42.2``  E 25º44`05.5`` | 8.2 | 32.1 | 0.1 | 4.6 | High | 71.9 |
| Suolijärvi | 205 | 1023 | 10 | 9 | N 61º7´50.5``  E 24º49`13.5`` | 13.7 | 15.3 | 0.1 | 10.9 | High | 5.3 |
| Ekojärvi | 74 | 1827 | 8 | 7 | N 61º11´51.3``  E 24º57`23.7`` | 9.7 | 1.2 | 0.03 | 20.7 | Low | 5.3 |
| Kataloistenjärvi | 112 | 267 | 2 | 1.9 | N 61º01´10.6``  E 25º56`44.0`` | 20.9 | 0.6 | 0.03 | 0.9 | Low | 202.8 |
| Teuronjärvi | 134 | 528 | 5 | 4 | N 61º03´41.9``  E 24º51`42.3`` | 17.6 | 0.7 | 0.1 | 61.4 | Low | 4.7 |
| Kyynäröjärvi | 25 | 758 | <3 | 2.5 | N 61º07´15.2``  E 24º59`32.6`` | 17.2 | 0.6 | 0.1 | 28.4 | Low | 6.6 |
| Kastanajärvi | 33 | 0 | 9 | 3 | N 61º13´41.4``  E 24º48`11.9`` | 19.4 | 0.6 | 0.03 | 0.6 | Low | 102.8 |
| Lehee | 105 | 110 | 2.5 | 2.2 | N 61º13´41.4``  E 24º48`11.9`` | 21.4 | 0.4 | 0.03 | 2.1 | Low | 210.0 |

* Jyväsjärvi is an urban lake
