# Supplementary material for: Genetic and Environmental Controls on Nitrous Oxide Accumulation in Lakes
Source: PLoS One. 2015 Mar 10;10(3):e0121201. doi: 10.1371/journal.pone.0121201 (PMC4355481; doi:10.1371/journal.pone.0121201)
Supplement: S2 Table — (DOCX) [file pone.0121201.s004.docx]

Table S2. Gene copy numbers of *nirS*, *nirK*, *nosZ*_I_, and *no*sZ_II_ gene amplicons per ng of DNA (nd, no data).

| Inter-lake  comparison | Gene copy number (ng^-1^ of DNA) | | | | High-nitrate/  Low-nitrate |
| --- | --- | --- | --- | --- | --- |
|  | *nir*S | *nirK* | *nosZ*_I_ | *nosZ*_II_ |  |
| Pääjärvi | 1.6x10^2^ | 2.3x10^2^ | 1.4x10^2^ | 6.4x10^1^ | High |
| Mommilanjärvi | 2.3x10^2^ | 1.2x10^2^ | 1.3x10^2^ | 1.2x10^2^ | High |
| Ormajärvi | 2.6x10^2^ | 2.8x10^2^ | 1.7x10^2^ | 1.5x10^2^ | High |
| Vanajavesi | 8.9x10^1^ | 1.0x10^2^ | 7.4x10^1^ | 4.1x10^1^ | High |
| Jyväsjärvi | 9.8x10^1^ | 1.1x10^2^ | 8.4x10^1^ | 1.7x10^2^ | High |
| Suolijärvi | 2.8x10^1^ | 4.4x10^1^ | 2.9x10^1^ | 5.1x10^1^ | High |
| Ekojärvi | 4.8x10^0^ | 9.6x10^0^ | 8.3x10^0^ | 1.1x10^1^ | Low |
| Kataloistenjärvi | 4.8x10^2^ | 5.8x10^2^ | 4.3x10^2^ | 1.4x10^2^ | Low |
| Teuronjärvi | 1.9x10^2^ | 2.3x10^2^ | 2.2x10^2^ | 1.1x10^2^ | Low |
| Kyynäröjärvi | 4.6x10^1^ | 6.7x10^1^ | 5.5x10^1^ | 4.7x10^1^ | Low |
| Kastanajärvi | 3.5x10^1^ | 2.6x10^1^ | 1.1x10^2^ | 2.3x10^2^ | Low |
| Lehee | 4.0x10^1^ | 7.8x10^1^ | 4.7x10^1^ | 8.3x10^1^ | Low |
| *Intra-lake*  *depth transect* |  |  |  |  |  |
| Vanajavesi2 | 5.2x10^2^ | 2.6x10^2^ | 2.2x10^2^ | 1.9x10^2^ |  |
| Vanajavesi3 | 3.3x10^2^ | 1.9x10^2^ | 1.8x10^2^ | 1.3x10^2^ |  |
| Vanajavesi4 | 2.5x10^2^ | 1.9x10^2^ | 1.7x10^2^ | 8.8x10^1^ |  |
| Vanajavesi5 | 1.0x10^2^ | 1.3x10^2^ | 9.1x10^1^ | 4.3x10^1^ |  |
| Vanajavesi6 | 8.3x10^1^ | 1.5x10^2^ | 8.4x10^1^ | 2.1x10^1^ |  |
| Vanajavesi7 | 8.9x10^1^ | 1.0x10^2^ | 7.4x10^1^ | 4.2x10^1^ |  |
| Vanajavesi8 | 1.7x10^2^ | 1.2x10^2^ | 6.5x10^1^ | nd |  |
