# Supplementary material for: Genetic and Environmental Controls on Nitrous Oxide Accumulation in Lakes
Source: PLoS One. 2015 Mar 10;10(3):e0121201. doi: 10.1371/journal.pone.0121201 (PMC4355481; doi:10.1371/journal.pone.0121201)
Supplement: S3 Table — (DOCX) [file pone.0121201.s005.docx]

Table S3. Water temperature and pH, as well as nutrient and oxygen concentrations at various sampling sites in Lake Vanajavesi

| Site | Coordinates | Depth (m) | O_2_  (µmol l^-1^) | Temperature (°C) | NO_3_  (µmol l^-1^) | PO_4_ (µmol l^-1^) | NH_4_ (µmol l^-1^) | pH |
| --- | --- | --- | --- | --- | --- | --- | --- | --- |
| Vanaja1 | N 61º08.07.8``  E 24º17`43.8`` | 3 | 221.6 | 20.3 | 24.0 | 0.03 | 1.1 | 7.5 |
| Vanaja2 | N 61º07´35.9``  E 24º17`19.5`` | 4 | 201.9 | 19.8 | 33.4 | 0.2 | 8.9 | 7.6 |
| Vanaja3 | N 61º07´53.4``  E 24º17`18.3`` | 6 | 243.4 | 20.1 | 32.9 | 0.3 | 15.8 | 7.7 |
| Vanaja4 | N 61º08´17.9``  E 24º16`59.4`` | 8 | 138.1 | 18.4 | 40.2 | 0.6 | 8.4 | 7.1 |
| Vanaja5 | N 61º08´28.4``  E 24º16`48.4`` | 10 | 65.9 | 16.3 | 36.3 | 0.3 | 23.5 | 6.9 |
| Vanaja6 | N 61º08´04.12``  E 24º16`38.6`` | 12 | 38.8 | 14.9 | 44.9 | 0.7 | 13.8 | 6.8 |
| Vanaja7 | N 61º08´58.7``  E 24º16`38.2`` | 14 | 1.9 | 13.5 | 39.9 | 0.6 | 27.3 | 7.0 |
| Vanaja8 | N 61º09´03.2``  E 24º16`11.1`` | 16 | 1.9 | 12.8 | 32.1 | 0.1 | 57.4 | 7.1 |
