# Supplementary material for: Genetic and Environmental Controls on Nitrous Oxide Accumulation in Lakes
Source: PLoS One. 2015 Mar 10;10(3):e0121201. doi: 10.1371/journal.pone.0121201 (PMC4355481; doi:10.1371/journal.pone.0121201)
Supplement: S4 Table — (DOCX) [file pone.0121201.s006.docx]

Table S4. Gene-specific primer pairs used in the qPCR assays

| Target gene | Primer sequence | Reference |
| --- | --- | --- |
| 16S rRNA 27f  16S rRNA 338r | 5’-AGAGTTTGATCMTGGCTCAG-3’  5’ -TGCTGCCTCCCGTAGGAGT-3’ | Bacterial primer (Lane 1991)  Universal primer |
| nirSCd3aF  nirSR3cd | 5’-AACGYSAAGGARACSGG-3’  5’-GASTTCGGRTGSGTCTTSAYGAA-3’ | Kandeler et al. 2006 |
| nirK876  nirK1040 | 5’-ATYGGCGGVAYGGCGA-3’  5’-GCCTCGATCAGRTTRTGGTT-3’ | Henry et al. 2004 |
| nosZ2F cladeI  nosZ2R cladeI | 5’-CGGRACGGCAASAAGGTSMSSGT-3’  5’-CAKRTGCAKSGCRTGGCAGAA-3’ | Henry et al. 2006 |
| nosZ-II-F cladeII  nosZ-II-R cladeII | 5’-CTIGGICCIYTKCAYAC-3’  5’-GCIGARCARAAITCBGTRC-3’ | Jones et al. 2013 |

References:

Henry S, Baudoin E, López-Gutiérrez JC, Martin-Laurent F, Brauman A and Philippot L (2004) Quantification of denitrifying bacteria in soils by *nir*K gene targeted real-time PCR. J Microbiol Meth 59: 327-335.

Henry S, Bru D, Stres B, Hallet S and Philippot L (2006) Quantitative Detection of the *nos*Z Gene, Encoding Nitrous Oxide Reductase, and Comparison of the Abundance of 16S rRNA, *nar*G, *nir*K, and *nos*Z Genes in Soils. Appl Environ Micorbiol 72 5181-5189.

Jones CM, Graft DRH, Bru D, Philippot L and Hallin S (2013) The unaccounted yet abundant nitrous oxide-reducing microbial community: a potential nitrous oxide sink. ISME J 7: 417-426.

Kandeler E, Deiglmayr K, Tscherko D, Bru D & Philippot L (2006) Abundance of narG, nirS, nirK, and nosZ Genes of Denitrifying Bacteria during Primary Successions of a Glacier Foreland. Appl Environ Microbiol 72: 5957–5962.

Lane DJ (1991) 16S/23S rRNA sequencing In: E Stackebrandt & M Goodfellow (ed) Nucleic acid techniques in bacterial systematic John Wiley & Sons pp115-175
